# A cryptic new species of *Chlidonoptera* Karsch, 1892 from the south west protected zone of the Central African Republic (Insecta, Mantodea, Hymenopodidae)

**DOI:** 10.3897/zookeys.917.39270

**Published:** 2020-03-09

**Authors:** Nicolas Moulin

**Affiliations:** 1 Institut Systématique, Evolution, Biodiversité (ISYEB), Muséum national d’Histoire naturelle, UMR 7205, MNHN, CNRS, Sorbonne Université, EPHE, Universités des Antilles, CP50, 45 rue Buffon, 75231 Paris Cedex 05, France Sorbonne Université Paris France

**Keywords:** Afrotropical, *
Chlidonoptera
*, cryptic species, DNA barcoding, praying mantis, taxonomy

## Abstract

Between 1998 and 2012, several scientific expeditions in Dzanga-Sangha Special Reserve and Dzanga-Ndoki National Park led to the collection of many Mantodea specimens from Central African Republic (CAR). Among these specimens, several males of an undescribed species were discovered. Morphologically, this species most closely resembles to *Chlidonoptera vexillum* Karsch, 1892 and *Chlidonoptera lestoni* Roy, 1975. A new lineage was revealed by DNA barcoding. Therefore, a new species is described, *Chlidonoptera roxanae***sp. nov.** Habitus images, genitalia illustrations and descriptions, measurement data, a key to species, natural history information, and locality data are provided. These results add to the evidence that cryptic species can be found in tropical regions, a critical issue in efforts to document global species richness. They also illustrate the value of DNA barcoding, especially when coupled with traditional taxonomic tools, in disclosing hidden diversity.

## Abstract (fr)

Entre 1998 et 2012, plusieurs expéditions scientifiques, dans la Réserve Spéciale de Dzanga-Sangha et dans le Parc National de Dzanga-Ndoki, ont permis de recueillir de nombreux spécimens de Mantodea en République centrafricaine (RCA). Parmi ceux-ci, plusieurs mâles d’une espèce non décrite ont été mis en évidence. Sur le plan morphologique, l’espèce est proche de *Chlidonoptera vexillum* Karsch, 1892 et de *Chlidonoptera lestoni* Roy, 1975. Le séquençage ADN a mis en lumière cette espèce. Par conséquent, une nouvelle espèce est décrite, *Chlidonoptera roxanae***sp. nov.** Des images des habitus, des illustrations et descriptions des genitalia, des données de mesure, une clé pour les espèces, des informations d’écologie et des données de localité sont fournies. Les résultats ajoutent à la preuve que les espèces cryptiques peuvent être trouvées dans les régions tropicales, un problème crucial dans les efforts visant à documenter la richesse en espèces de la planète. Ils illustrent également la valeur du séquençage ADN, en particulier lorsqu’il est associé à des outils taxonomiques traditionnels, pour la mise en évidence de la diversité cachée.

## Introduction

Since the beginning of the 1980s, the entomologist Philippe Annoyer had been traveling in southwestern CAR searching for butterflies and other insects. In 2008, his missions expanded and came to be called Epiphyte 2008. In 2010, a massive survey program was organised under the name SANGHA2012 Biodiversité en Terre Pygmée. On this occasion, the author joined the team to increase the study and collections of Mantodea (Moulin et al. 2017, Moulin 2018b). Several males of *Chlidonoptera* Karsch, 1892 were collected, mostly by light trapping. Visual searching and beating of vegetation, both on the ground and canopy, did not lead to the discovery of the associated female. The localities of these specimens are in the last remnants of primary forests of the southwestern tip of the CAR.

All species belonging to the genus *Chlidonoptera* are morphologically similar to each other but easily discriminated from other genera. The main morphological feature of the genus is a relatively large yellow spot on the elytra located between the two black arcs of the circle. The collected male *Chlidonoptera* specimens were initially presumed to be *C. vexillum* Karsch, 1892, as they share many morphological similarities. Additional examinations of *C. vexillum* male genitalia compared to the recently collected *Chlidonoptera* genitalia led to the submission of DNA sequencing samples at Canadian Center for DNA Barcoding (CCDB) in Guelph. Many studies have used the 5’ region of the cytochrome oxidase I gene (COI), more commonly referred to as the DNA barcode region, as a useful tool to discriminate various groups of insects (Cocuzza et al. 2015). The results from the DNA sequencing revealed that the specimens from southwestern CAR are different from known *C. vexillum* Karsch, 1892 specimens from Cameroon and Gabon. Originally described by Karsch in 1892 (*Bomistria lunata* Saussure, 1898 synonym) to contain a single species *C. vexillum*, two species were added: *C. chopardi* Roy, 1964 and *C. lestoni* Roy, 1975. During this time, Roy synonymised the East African *Anabomistria werneri* Giglio-Tos, 1915 (Roy 1964) with *Chlidonoptera*, which was later confirmed by Lombardo (1997). Thus, prior to the discovery of this new species, described herein, the genus *Chlidonoptera* contained four species: *C. vexillum*, *C. chopardi*, *C. lestoni*, and *C. werneri*.

*Chlidonoptera chopardi* is distributed in West Africa, *C. vexillum* and the new species are distributed in West Central Africa, *C. lestoni* is distributed in Ghana (Leston 1968, Roy et Leston 1975), with *C. werneri* distributed in the East. It appears that Tanzania and Kenya are the eastern limits of the distribution of *C. vexillum* (Ehrmann 2002, Schwarz and Roy 2019). *Chlidonoptera vexillum* is sympatric with *C. werneri*, creating confusion. Wrongly, Kirby (1904) cites *Bomistria lunata* Saussure, 1898, as a distinct species of *C. vexillum*. *Chlidonoptera* is classified within the tribe Hymenopodini, subtribe Pseudocreobotrina with four other genera (Mantodea Species File, http://mantodea.speciesfile.org; Svenson et al. 2016, Schwarz and Roy 2019).

Ideally the description of a species should result from a synthesis of information that encompasses morphological, molecular, biological, biogeographical, physiological, ecological and bibliographical data; however, this compendium of information is lacking for the great majority of species.

## Materials and methods

### Sampled region

The study area includes the UNESCO World Heritage site Sangha Trinational, the Dzanga-Sangha Special Reserve (6,865.54 sq km) and the Dzanga-Ndoki National Park (1,143.26 sq km) (Moulin et al. 2017). These national parks and reserves aim to protect the second largest rain forest on the earth. Altitude ranges from 300 to 620 meters above sea level. The whole zone is on alluvial sands. Along streams, forest clearings are present with marshy depressions. There are three types of forest within the study area: mainly dryland forest, a semi-evergreen forest that contains swamp-forest areas along the rivers, and a closed-canopy, monodominant *Gilbertiodendron dewevrei* forest. The dryland forest is an open, mixed canopy that is dominated by Sterculariaceae and Ulmaceae; often associated with it is a dense understory of Marantaceae and Zingiberaceae. Along the Sangha river, there are stands of *Guibourtia demeusei* (Vande Weghe 2004, http://www.dzanga-sangha.org/).

### Collection and preparation

Collection was predominately made by light trapping with 250-Watt bulbs. A few individuals were found at or around the lamp; or on the tents of the camp, attracted by the diffuse light of the incandescent bulbs. The specimens were placed in cyanide vials and then kept dry on layers of cotton and blotting paper. Some specimens were kept alive in cubital screen enclosures to capture live images. Some males were pinned after genitalia preparation was made and a leg was preserved in ethanol for DNA barcoding with tissue samples deposited in CCDB in Guelph.

### DNA barcoding

DNA barcoding, the analysis of a standardised segment of the mitochondrial cytochrome c oxidase subunit I (COI) gene, was performed on a representative selection of specimens (n = 25). Tissues were sent to CCDB at the University of Guelph for DNA extraction, polymerase chain reaction (PCR), and sequencing. DNA was extracted from dry legs using a routine silica-based 96-well extraction automation protocol (Ivanova et al. 2006). The 658bp region of COI proposed for use as a ‘DNA barcode’ (Hebert et al. 2003) was amplified with the PCR primers C_LepFolF/C_LepFolR (Hebert et al. 2004). Data are currently managed under the following projects: “Mantodea of Gabon – Project 1 [ECOTROP 2014],” “Mantodea of Gabon – Project 2 [ECOTROP 2011],” “DNA Barcoding Mantodea - Collection N. Moulin” Barcode of Life Data Systems (BOLD, Biodiversity Institute of Ontario, Canada; http://www.boldsystems.org). Kimura-2-parameter (K2P) distances were calculated using the BOLD 4.0 interface (Ratnasingham and Hebert 2007). Sequences were then analysed and trees constructed using the BOLD 4.0 interface.

### Deposition of the specimens

Specimens are, currently, in the Research Collection of Nicolas Moulin (Montérolier, France) and Philippe Annoyer Personal Collection (Sainte-Croix-Volvestre, France). Types will be deposited at the MNHN (Paris, France).

Abbreviations used in this paper:

**BOLD** Barcode of Life Project, Biodiversity Institute of Ontario;

**RCNM** Research Collection of Nicolas Moulin, Montérolier;

**PAPC** Philippe Annoyer Personal Collection;

**DNNP** Dzanga-Ndoki National Park, Central African Republic;

**DSSR** Dzanga-Sangha Special Reserve, Central African Republic;

**MNHN**Muséum national d’Histoire naturelle, Paris.

### Descriptive conventions and character systems

The species treatment within this study provides a brief diagnosis and criteria descriptions stemming from the anterior surface of the head, the dorsal surface of the pronotum, the legs, the wings, and the abdomen. Foreleg spine nomenclature follows Wieland (2008, 2013) and morphological terminology, including genitalia, follows that of Brannoch et al. (2017) where diagrams of spine arrangements can be viewed.

**Measurements.** Specimens were measured using a Leica S8APO stereomicroscope with a caliper. All measurements in this study were taken with a caliper and are expressed in millimetres. A total of 22 measurement classes were captured, as in Tedrow et al. (2014), including:

1. Body length = length of body from central ocelli to posterior tip of abdomen (intraspecifically variable measurement, primarily for general size estimation).

2. Forewing length = from proximal margin of axillary sclerites to distal tip of the discoidal region.

3. Hindwing length = from proximal margin of axillary sclerites to distal tip of the discoidal region.

4. Pronotum length = from anterior margin to posterior margin.

5. Prozone length = anterior margin of pronotum to center of supra-coxal sulcus.

6. Pronotum width = from the lateral margins at the widest point, the supra-coxal bulge.

7. Ratio pronotum = ratio between pronotum width and length.

8. Pronotum narrow width = from lateral margins of the pronotum at the narrowest region of metazone.

9. Head width = from lateral margins of the eyes at the widest point.

10. Frons width = from lateral margins of the frons, inferior to the antennal insertions, at the widest point.

11. Frons height = from upper margin abutting central ocellus to lower margin abutting clypeus.

12. Prothoracic coxae length = from pronotum to trochanter.

13. Prothoracic femur length = from proximal margin abutting trochanter to distal margin of genicular lobe.

14. Mesothoracic femur length = from most proximal margin abutting the trochanter to the distal side of the terminal spine insertion site.

15. Mesothoracic tibia length = from most proximal groove near joint with the femur to the distal side of the terminal spine insertion site.

16. Mesothoracic tarsus length = from proximal joint to the apex of the ungues curve.

17. Metathoracic femur length = from most proximal margin abutting the trochanter to the distal side of the terminal spine insertion site.

18. Metathoracic tibia length = from most proximal groove near femoral joint to the distal side of the terminal spine insertion site.

19. Metathoracic tarsus length = from proximal joint to the apex of the ungues curve.

20. Anteroventral femoral spine count = all inner marginal ridge spines, except the distal terminal spur.

21. Anteroventral tibial spine count = all inner marginal ridge spines, except the distal terminal spur.

22. Posteroventral tibial spine count = all outer marginal ridge spines but except the distal terminal spur.

The measurement of the total body length produces a measurement only useful for general assessment of body size rather than species description. Since head position, abdominal expansion, and wing position are all variable, total body length should only be used as a rough measurement to initially discriminate between the small and large Mantodea species when performing identifications.

**Imaging.** Alive specimen was captured with a NIKON D700 by Philippe Annoyer on 3 December 2010 near the base camp in Dzanga-Ndoki NP. Habitus images were taken with a Konica Minolta Dynax 5D. All images were taken over an 18% grey card background for white balance standards, excluding the image of the *C. lestoni* paratype from the MNHN. Images were processed in GIMP 2 to adjust levels, contrast, exposure, sharpness, and to add scale bars. Minor adjustments were made using the stamp tool to correct background aberrations and to remove distracting debris. Plates were constructed using Publisher 2016.

## Taxonomic placement

The following characters led to place the new species within *Chlidonoptera* genus: mantids of medium size and bright colours, very similar to *Pseudocreobotra* genus; but the tips of the lower frons and clypeus very short and blunt, the protuberance of the vertex shorter. The eyes are bulging but rounded. Less expanded pronotum, shorter than anterior coxa: prozone more compressed, higher with two acute conical tubers in front of supracoxal sulcus, no tubercles on the metazone. Wings are beyond the abdomen in both sexes. Forewings of females more dilated from base to apex and hindwings almost opaque, yellow with dark veins; males only the basal part with this coloration, the rest hyaline. Forewings with a large eye spot, a yellow spot near the shoulder and apex on light colour. Anterior femurs are thin. The external spines of the anterior coxa are not swollen at the base, four discoidal spines and four posteroventral femoral spines. Femurs of the meso- and metathoracic legs have a subapical and posteroventral lobe. Laterally lobed present on the abdominal segments.

Known species of the genus *Chlidonoptera* were compared to the males found in southwestern CAR. Distribution of known individuals of *C. werneri*, the structure of the genitalia and the morphology described in Roy (1964) and Lombardo (1997) exclude it as a candidate species. Similarly, distribution, structure of the genitalia and morphology described in Roy (1964) excluded *C. chopardi* as the species. On the other hand, the distinction between *C. vexillum* and *C. lestoni* is much more complicated (Roy and Leston 1975; for reference, the imaged types can be seen at http://specimens.mantodea.com). Morphologically, the three species are very similar. Only the structure of the posterior end of the sclerite L4A of the ventral phallomere (hypophallus) enables to distinguish them. The COI-DNA barcoding of 19 *Chlidonoptera* specimens enabled the differentiation of the new species from *C. vexillum* collected in Gabon (Moulin 2018a) and Cameroon.

### Chlidonoptera

Taxon classificationAnimaliaMantodeaHymenopodidae

Karsch, 1892

BA9BA587-7F91-52CB-B0A7-683A19FE55F8

Chlidonoptera : Karsch 1892: 68; Karsch 1892: 150; Karsch 1894: 278; Saussure 1898: 789; Kirby 1904: 292; Giglio-Tos 1927: 563; Beier 1934: 26; Beier 1964: 939; Roy 1964: 764; Roy 1965: 595; Ragge and Roy 1967: 634; Beier 1968: 6; Roy 1975: 163; Roy and Leston 1975: 329; Ehrmann 2002: 95; Otte and Spearman 2005: 86; Svenson et al. 2016: 6; Schwarz and Roy 2019: 151.

#### Type species.

*Chlidonoptera vexillum* Karsch, 1892.

#### Taxonomic history.

Fred Karsch created the genus *Chlidonoptera* in 1892 (p. 68) for two females specimen collected by Dr. P. Preuss in Cameroon, at Buea, *C. vexillum* Karsch, 1892. Karsch (1892: 150) cited *C. vexillum* from the collections of Dr P. Preuss in Cameroon, with a relatively detailed description of female types from Buea. In a new list of Mantodea collected by Dr P. Preuss in Cameroon, Karsch (1894: 278) for a third time cited the two females from Buea, with an illustration of a female at the end of the document. H. de Saussure created the genus *Bomistria* in 1898 (pg. 202) for a male specimen from Gabon, *B. lunata* Saussure, 1898. In 1900, Y. Sjöstedt (pg. 20) gave measurements for females of *C. vexillum* and males of *B. lunata*, without putting them in synonymy. The genus was then misspelled, ‘*Clidonoptera*.’ W.F. Kirby (1904: 292) continued to conserve the two species, *C. vexillum* and *Bomistria lunata*, with also a misspelling in the Sjöstedt citation, ‘*Chlinidonoptera*.’ F. Werner (1908: 52), making the point between *Chlidonoptera vexillum* and *Bomistria lunata* with supporting illustrations. But, in 1915, Giglio-Tos clarified the situation: *B. lunata* of Saussure is the male of *C. vexillum* and as the female *B. lunata* of F. Werner would be a new genus with a new species, *Anabomistria werneri* Giglio-Tos, 1915. The location of *A. werneri* was listed only as ‘Africa’ (Giglio-Tos 1927: 563). In his great synthesis work, *Genera Insectorum*, Beier (1934: 26) listed *C. vexillum* and *A. werneri* with a description of their morphological features. He stated that *A. werneri* is from East Africa. Then, in 1964 (p. 939), he confirmed the locality of these species in Hymenopodidae and Hymenopodinae. That same year, R. Roy (1964) synthesised data about Mantodea from the Ivory Coast forest, wherein a new species of *Chlidonoptera* was described, *C. chopardi* (p. 764); the male genitalia of which were compared with those of *C. vexillum*. At the same time, the author reconsidered the genus *Anabomestria* and logically placed *A. werneri* in the genus *Chlidonoptera*. M. Beier (1968: 6, fig. 6b) illustrated the right forewing of *A. werneri*’s female but the taxonomic change of genus made by Roy four years earlier was not taken into account. Later, *Chlidonoptera lestoni* was described (Roy and Leston 1975: 329) from Ghana. In that same work, *C. chopardi* was also cited. A comparison of the posterior process (pda) of the ventral phallomere was illustrated for *C. chopardi*, *C. lestoni*, and *C. vexillum*. It was assumed that *C. lestoni* was close to *C. vexillum* but distinct; this was not like that which D. Leston wrote in 1968. F. Lombardo (1997: 80) completed the description of *C. werneri* with a male specimen collected from Tanzania. Finally, R. Ehrmann (2002: 96) summarised all that was known about *Chlidonoptera* and D. Otte & L. Spearman did the same in 2005 (p 86).

### Identification key to species of *Chlidonoptera* using males

The key to the morphological criteria of *Chlidonoptera* species can only distinguish *C. chopardi*, *C. werneri* and a complex of species, named *vexillum group*, including *C. vexillum*, *C. lestoni* and *C. roxanae* sp. nov.

**Table:** 

1	The smallest species, 23–26 mm (male); Prolongation of the vertex non bifid; forewings with yellow costal area; green discoidal area on almost two-thirds of the basal area, with two yellow spots and two black arcs as in *vexillum group*, but closer together; hindwings hyaline with pink coloured base; the posterior process of the ventral phallomere smaller and thin	***C. chopardi***
–	Larger species, 24–34 mm (male), 37–40 mm (female); prolongation of the vertex bifid, with a more or less notched summit; two black arcs on forewings more separated than in *C. chopardi*	**2**
2	Lateral margins of the pronotum smooth; largest anteroventral femoral spines black; wings uniformly yellowish white	***C. werneri***
–	Lateral margins of the pronotum finely granular; yellowish hind wings with red-brown veins from the anal area and extending variably until the first third of the wing	***vexillum group***

The three species of the *vexillum group* are difficult to differentiate without using male genitalia. There is a size gradient of the posterior process of the ventral phallomere from the smallest to the largest, from *C. lestoni* to *C. roxanae* sp. nov. through *C. vexillum*, in proportion to the body size. Genitalia of *C. lestoni* and *C. vexillum* are represented in Roy & Leston (1975: fig. 9) and in Roy (1964: fig. 7).

**Figure 1.**
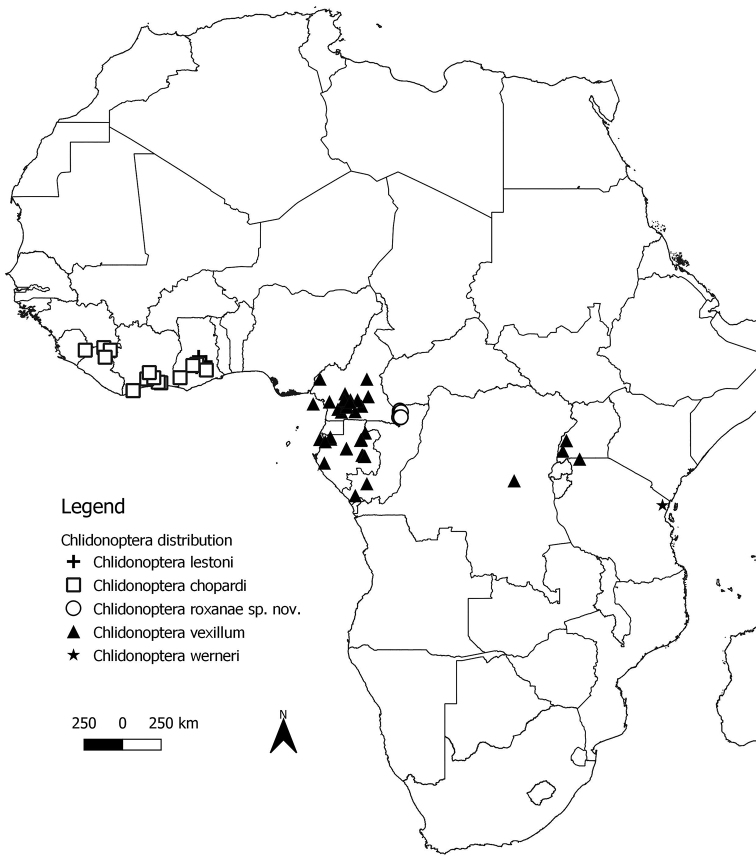
Distribution map of *Chlidonoptera* species. Source: http://www.gadm.org Global Administrative areas Data and Maps (GADM).

The distributions of the different species of *Chlidonoptera* are shown on the map in Figure 1.

### Chlidonoptera vexillum

Taxon classificationAnimaliaMantodeaHymenopodidae

Karsch, 1892

E771C1EA-BE58-50EE-861C-1A45F6B17868

Figure 4

Chlidonoptera vexillum : Karsch 1892: 68; Karsch 1892: 150; Karsch 1894: 279; Sjostedt 1900: 20; Beier 1934: 27; Roy 1973: 235; Ehrmann 2002: 95; Otte and Spearman 2005: 87.
= Bomistria lunata: Saussure 1898: 789; Kirby 1904: 292; Giglio-Tos 1927: 563; Beier 1934: 26; Ehrmann 2002: 95; Otte and Spearman 2005: 87.

#### Material examined.

(5♀♀, 100♂♂). **Cameroon.** Doumé (1♀), 1930, Coll. M. Cazal, MNHN; Locality unknown (1♂), 1934, Coll. P. Magnier, genitalia preparation Roy 220, MNHN; Edea (1♂ 1♀), VIII.1956, Collector M. de Lisle, genitalia preparation Roy 221, MNHN; Nkolbisson, 30.VI.1965 (1♂) & 24.XII.1969 (1♂), Coll. B. de Miré, MNHN; Kala (5♂♂), 25.XI.1972 to II.1973, Coll. Ph. Darge, genitalia preparation Roy 2074, 2080, and 2082, MNHN; Dokoa, savannahs and forest galleries of Sanaga (1♂), 12.X.1973, Coll. Ph. Darge, MNHN; Kala, Nkolbiyong Mountain, 1150 m (4♂♂), 20.X.1973, Coll. Ph. Darge, MNHN; Ayos, banks of Nyong, 13 km NNW of Obaut, 04.V.1973 (1♂) and 15 to 25.XI.1973 (1♂), Coll. Ph. Darge, MNHN; Elang, 140 km SSE of Yaoundé (1♂), V.1974, Coll. Ph. Darge, MNHN; Mbam-Minkom, Nouma Mountain, 12 km NNW of Nkolbisson, 1000m (1♂), XII.1974, Coll. Ph. Darge, MNHN; Dzeng Forest, 650 m (11♂♂), 10 to 20.III.1975, Coll. Ph. Darge, genitalia preparation Roy 2203, MNHN; Mbitom (1♂), 20.IV.1975, Coll. Ph. Darge, MNHN; Ngom, banks of Soo (6♂♂), I.1976, Coll. Ph. Darge, MNHN; Nkolmélié, banks of Nyong (1♂), 25.I.1976, Coll. Ph. Darge, MNHN; Nemeyong (1♂), 25.II.1976, Coll. Ph. Darge, MNHN; Meukowong (4♂♂), III.1976, Coll. Ph. Darge, MNHN; Fakélé (#2), 660 m (3♂♂), 20 to 25.X.1976, Coll. Ph. Darge, MNHN; Mbio, Mamfe region (2♂♂), 1 to 5.VI.1977, Coll. Ph. Darge, MNHN; Bioko (1♀), VI.1997, Coll. Canu, MNHN; Center, South, Light Trap (1♂), 01.X.1998, Coll. Desfontaine, BOLD LopeMAN14-063, Genitalia NM0156, RCNM; Mbalmayo, Mfou Village, 750 m, Light Trap (1♂), XII.2013, Coll. Ph. Le Gall, BOLD NMMAN11-0541, RCNM.

**Central African Republic.** ‘Congo français, Haute-Sanga’ (1♀), 106-97, Coll. P.A. Ferrière, MNHN.

**Democratic Republic of the Congo.** Maniéma, Kindu (1♀), 1917, Coll. L. Burgeon, MNHN.

**Gabon.** Belinga, Mission biologique (3♂♂), 19.III.1963, Coll. H. Coiffait and before 1964, Coll. P. Grassé, MNHN; Plateau d’Ipassa (8♂♂), 27.X to 06.XII.1967, Coll. G. Bernardi, MNHN; Komo, Cristal mountains foothills, 400 m (3♂), 01 to 15.X.1969, Coll. A. Villiers, MNHN; Mvoum, Montagne de sable (1♂), 01 to 15.XI.1969, Coll. A. Villiers, MNHN; Makokou, Ipassa (4♂♂), 02 to 30.V.1971, Coll. J. Mateu, MNHN; Makokou, Balachowsky-Menier Mission (1♂), 29.XI.1973, Coll. A. Balachowsky, MNHN; Cristal Mountains NP (1♂), 24.VI.1993, Coll. E. Cherlonneix, MNHN; Ogooue-Maritime, Abanda caves, Light Trap (1♂), 06.VIII.2010, Coll. Th. Decaëns & D. Sebag, Genitalia NM0157, MNHN; Ogooue-Ivindo, Lope NP, Lope 2, Light Trap (2♂), 27.II.2011, Coll. Th. Decaëns & R. Rougerie, BOLD Lope11-0208 & 0209, Genitalia NM0158 & 0159, RCNM; Makokou (2♂), 14/20.IV.2012, Coll. G. Robiche, BOLD MANGAB15-090, MNHN; Ogooue-Ivindo, Lope NP, Panther Bridge, Remote Canopy Trap (1♂), 04.IV.2014,Coll. N. Moulin & G. Duvot, BOLD LopeMAN14-064, RCNM; Estuaire, Mondah, Arboretum Raponda Walker, Light Trap (2♂), 01.VI.2016, Coll. T. Decaëns, BOLD MANGAB15-094 & 095, RCNM; Ogooue-Lolo, Lastourville, Bambidie (13♂), 04/11.XI.2018, Coll. T. Decaëns & R. Rougerie, BOLD NMMAN11-0535, -0536, -0537, -0538, -0539, -0540, RCNM.

**Republic of the Congo.** M’Bila (1♂), XII.1963, Coll. A. Villiers, MNHN; Dimonika (1♂), 11.XI.1975, Coll. C. Morin, MNHN; Mayombe, Dimonika, Light Trap (1♂), 14.XI.1992, Coll. Ph. Le Gall, BOLD NMMAN11-0487, Genitalia NM0191, RCNM.

**Tanzania.** Kagera Region, Minziro Forest, 1160 m (1♂), 23.X.2010, Coll. Ph. Darge, BOLD NMMAN11-0533, ‘Museum de Lyon’.

**Uganda.** Kamwenge District, Kibale Forest, Chimp nest, Bigodi, 1240 m (2♂), 08.XI.2010, Coll. P. Schmit, BOLD MANGAB15-088, MNHN; Bushenyi District, Kalinzu Forest, Kitozho, 1450 m (1♂), 10.XI.2010, Coll. P. Schmit, MNHN; Kamwenge District, Kibale NP, Mainaro, 1260 m (2♂), 22.III.2012, Coll. P. Schmit, BOLD MANGAB15-089, MNHN.

### Chlidonoptera werneri

Taxon classificationAnimaliaMantodeaHymenopodidae

(Giglio-Tos, 1915)

02EF3506-A251-5830-B138-E81813803A9F

Anabomistria werneri : Giglio-Tos 1915: 108; Beier 1934: 26; Beier 1968: 6.
Chlidonoptera werneri : Roy 1964: 767; Lombardo 1997: 6; Ehrmann 2002: 95; Otte and Spearman 2005: 87.

### Chlidonoptera chopardi

Taxon classificationAnimaliaMantodeaHymenopodidae

Roy, 1964

148627A2-DB42-596E-94ED-1539BDDCB55D

Figure 4

Chlidonoptera chopardi : Roy 1964: 764; Roy 1965: 595; Ragge and Roy 1967: 586; Gillon and Roy 1968: 1039; Roy and Leston 1975: 297; Ehrmann 2002: 95; Otte and Spearman 2005: 86.

#### Type material examined.

(4♂♂). *Chlidonoptera chopardi*: Male holotype, Banco Forest Reserve, Ivory Coast, 1945, code “Ab 31 nuit,” Coll. R. Paulian & C. Delamare, genitalia preparation Roy 222, Insects – Small orders & Odonates MNHN Database (EP) #2329, MNHN; 1 ♂ paratype, Banco Forest Reserve, Ivory Coast, 1945, code “Ab 31 nuit,” Coll. R. Paulian & C. Delamare, Insects – Small orders & Odonates MNHN Database (EP) #2330, MNHN; 2 ♂ ♂ paratypes, Daloa, Ivory Coast, XII.1930/IV.1931, Coll. Ch. Alluaud & P. A. Chappuis, Insects – Small orders & Odonates MNHN Database (EP) #2331 & #2333, MNHN; 1 ♂ paratype, near Dimbokro, Ivory Coast, 1910, Coll. Capitaine Posth, Insects – Small orders & Odonates MNHN Database (EP) #2332, MNHN.

#### Other material examined.

(7♂♂) **Ivory Coast.** San Pedro (7♂), 05.XI.1982, Coll. Ph. Le Gall, Genitalia NM0160, 0161, 0162, RCNM.

### Chlidonoptera lestoni

Taxon classificationAnimaliaMantodeaHymenopodidae

Roy, 1975

BA9340F6-407E-5DF3-9A7A-52703716E9FE

Figure 4

Chlidonoptera lestoni : Roy 1975: 297; Ehrmann 2002: 95; Otte and Spearman 2005: 87.

#### Type material examined.

(1♂). *Chlidonoptera lestoni*: 1 ♂ paratype, Tafo, Ghana, 09.XI.1967, UV Trap, Coll. D. Leston, genitalia preparation Roy 2067, Insects – Small orders & Odonates MNHN Database (EP) #2488, MNHN.

### Chlidonoptera roxanae sp. nov.

Taxon classificationAnimaliaMantodeaHymenopodidae

6C187340-071C-5098-8254-E29609978C82

http://zoobank.org/E7FBAAE9-E506-4154-A762-3008A1D6AF44

**Figure 2.**
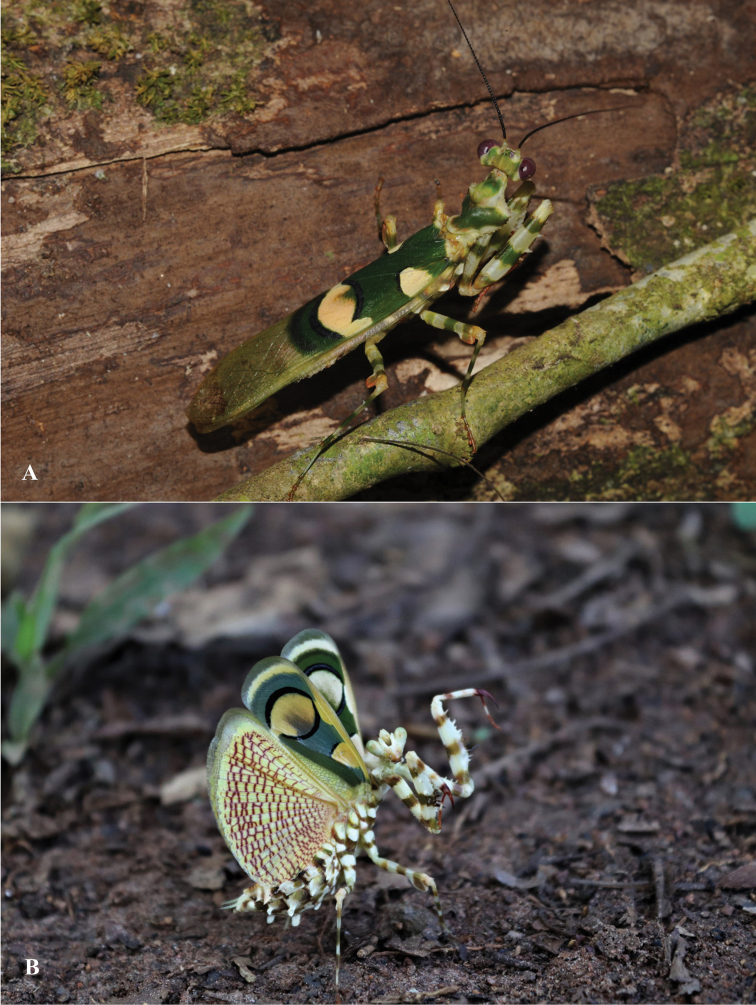
**A** Male *Chlidonoptera roxanae* sp. nov. photographed in the Dzanga-Ndoki National Park (CAR), by Philippe Annoyer **B** female *Chlidonoptera vexillum* group photographed in the forest surrounding Sanaga Yong Chimpanzee Rescue Centre, Belabo, East Province (Cameroon), by Sean Brogan.

**Figure 3.**
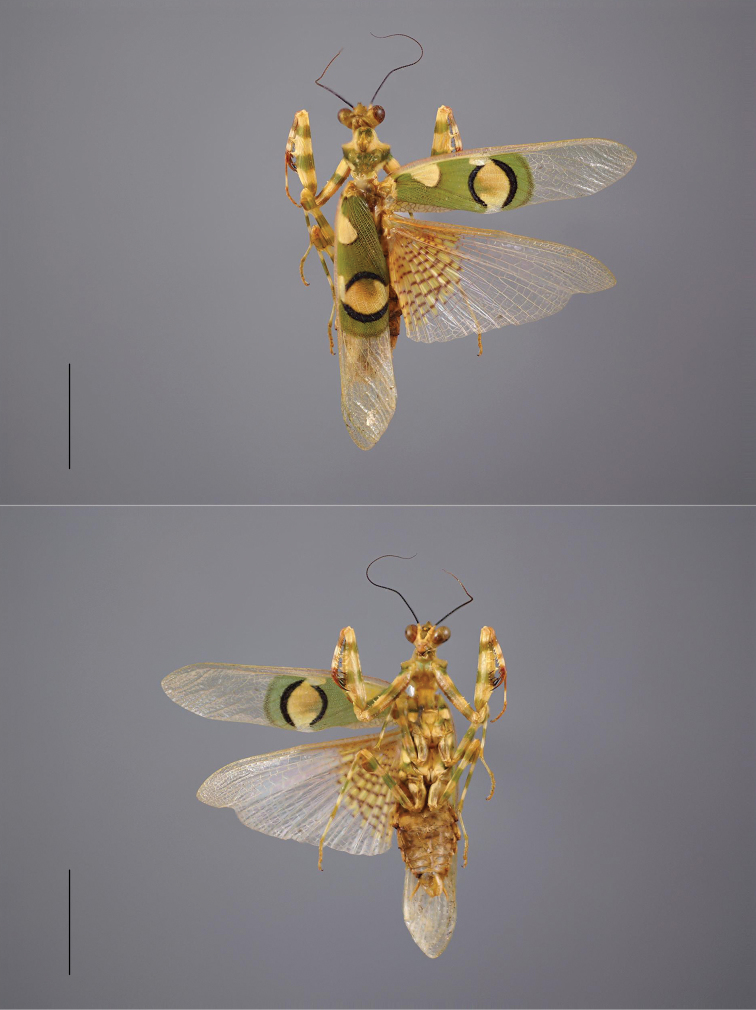
*Chlidonoptera roxanae* sp. nov., holotype male, dorsal and ventral habitus. Scale bar: 10.00 mm.

**Figure 4.**
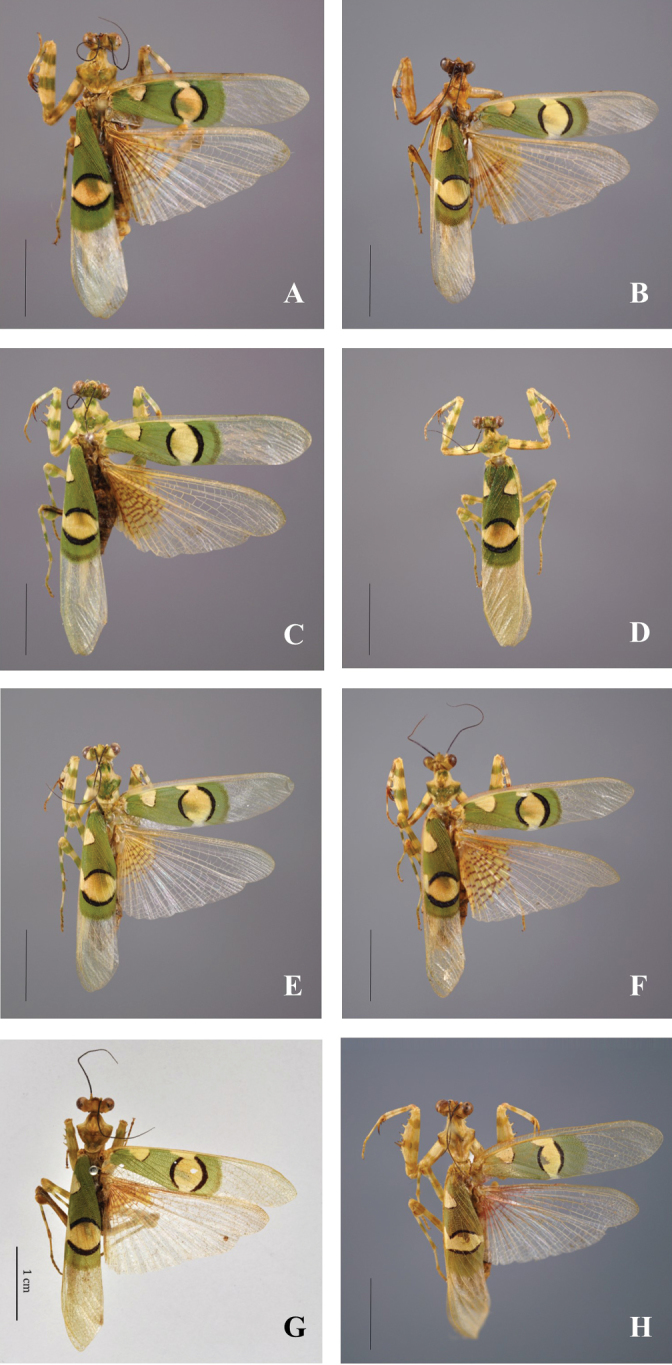
*Chlidonoptera*, dorsal habitus: **A***C. vexillum*, male, Mbalmayo, Cameroon, BOLD NMMAN11-0541 **B***C. vexillum*, male, Arboretum Raponda Walker, Gabon, BOLD MANGAB15-094 **C***C. vexillum*, male, Biosphere Reserve of Dimonika, Republic of the Congo, BOLD NMMAN11-0487 **D***C. vexillum*, male, Minziro Forest, Tanzania, BOLD NMMAN11-0533 **E***C. vexillum*, male, Kalinzu Forest, Uganda **F***C. roxanae* sp. nov., holotype male, base camp, lake #1, Dzanga-Ndoki NP, CAR, BOLD NMMAN11-0404 **G***C. lestoni*, paratype male, Tafo, Ghana (S. Poulain) **H***C. chopardi*, male, San Pedro, Ivory Coast.

**Figure 5.**
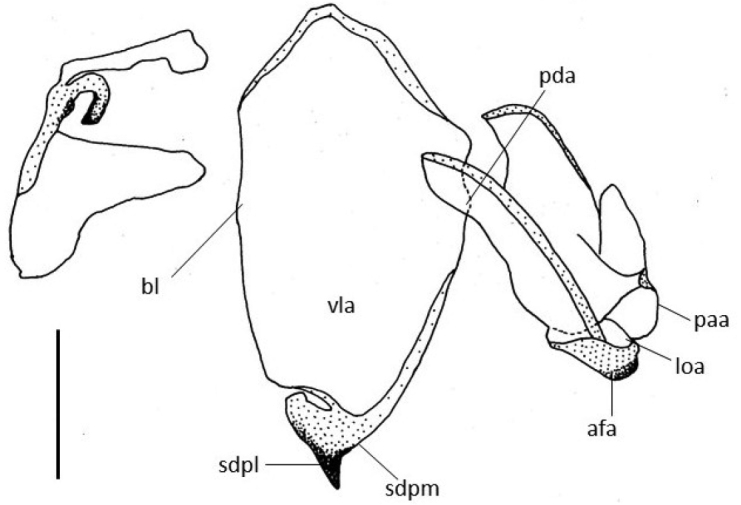
*Chlidonoptera roxanae* sp. nov., holotype male, Genitalia. afa = phalloid apophysis; paa = apical processof left phallomere, titillator; bl = basal lobe of ventral phallomere; loa = membranous lobe; pda = primary distal process; sdpl = lateral secondary distal process; sdpm = median secondary distal process; **vla** = ventral lobe of ventral phallomere. Scale bars: 1.00 mm.

**Figure 6.**
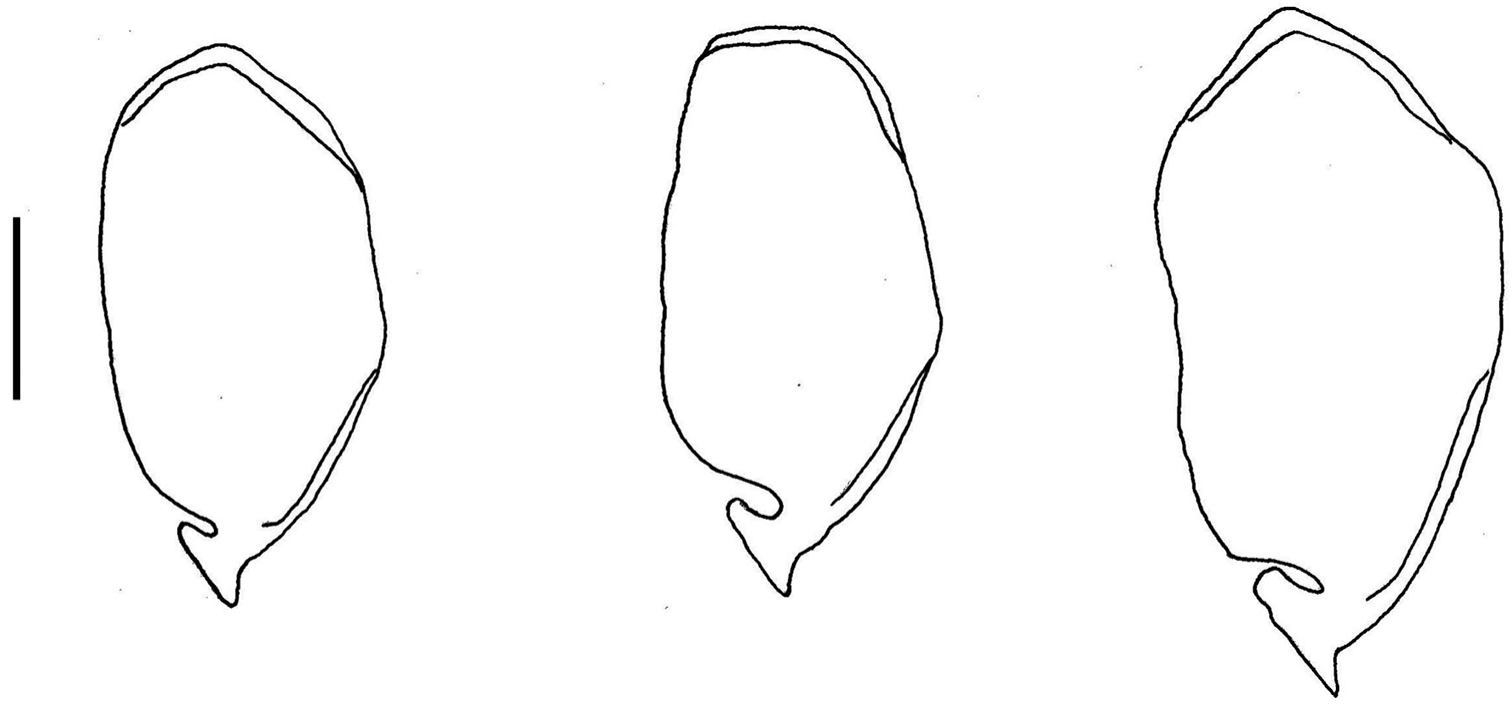
Differences between genitalia: left to right, *C. lestoni*, *C. vexillum*, and *C. roxanae* sp. nov. Scale bar: 1.00 mm.

Figures 2A , 3 , 4F , 5 , 6

#### Repository.

Holotype male. Muséum national d’Histoire naturelle, Paris, France.

Holotype label: Pinned. Central African Republic, Dzanga-Ndoki National Park, base camp, Lake #1, 2.4881, 16.2330, light, 4.II.2012, BOLD NMMAN11-0404, Genitalia NM0181, Coll: Sangha 2012 Team.

Paratypes males. Philippe Annoyer Personal Collection (PAPC), Sainte-Croix-Volvestre, France; Research Collection of Nicolas Moulin (RCNM), Montérolier, France; Muséum national d’Histoire naturelle, Paris, France.

#### Paratypes labels (28♂♂).

**Central African Republic.** Dzanga-Sangha Special Reserve, Bayanga, WWF building, diffuse light (1♂), 2.920333, 16.255527, 21.I.2012 (RCNM); Dzanga-Ndoki National Park, M’Boki, South Likembe, Molongo, Sangha river, light (1♂), 2.471972, 16.08125, 25.I.2012 (RCNM); M’Boki, South Likembe, Molongo, Sangha river, light (1♂), 2.471972, 16.08125, 25.I.2012 (MNHN); Base camp, Lake #1, windfall tree, light (7♂♂), 2.477916, 16.217388, 1–4.II.2012 (RCNM); Base camp, Lake #1, windfall tree, light (1♂), 2.477916, 16.217388, 1.II.2012 (MNHN); Lake #7, at the base of a Badamier (*Terminalia superba*, Combretaceae), light (1♂), 2.463277, 16.224833, 3.II.2012 (MNHN); Lake #1, at canopy of an Azobe (*Lophira alata*, Ochnaceae), light (1♂), 2.4804, 16.2155, 5.II.2012 (RCNM); Lake #1, base camp, windfall tree, laboratory tent, light (10♂♂), 2.480555, 16.216666, 10.II to 2.III.2012 (RCNM); Lake #3, light (2♂♂), 2.488611, 16.232944, 15 and 22.II.2012 (RCNM); at canopy of an Ayous (*Triplochiton scleroxylon*, Malvaceae), light (1♂), 2.488138, 16.233027, 22.II.2012 (MNHN); at canopy of an Ayous (*Triplochiton scleroxylon*, Malvaceae), light (1♂), 2.488138, 16.233027, 24.II.2012 (RCNM); Lake #7, light (1♂), 2.4806, 16.2167, 29.II.2012 (RCNM), Coll. SANGHA2012 Team.

#### Other material examined.

**Central African Republic.** Dzanga-Sangha Special Reserve, between Bayanga and Lidjombo, pk15 (2♂♂), pk21 (5♂♂), light, 2.883333, 16.254722, 31.V to 16.VI.1998 (PAPC), Coll. P. Annoyer; Dzanga-Ndoki National Park, Lidjombo (9♂♂: light (8♂♂) and day capture (1♂)), 2.833833, 16.137138, 1–13.III.2005 (PAPC), Coll. P. Annoyer; Dzanga-Sangha Special Reserve, Bayanga, base camp 1, light (2♂♂), 3.066194, 16.149888, 11.X.2008 (PAPC); Bayanga, base camp 2, night capture (1♂), 3.030416, 16.142138, 20.X.2008 (PAPC); Bayanga, at the base of a Kungu (*Piptadenastrium africanum*, Fabaceae) (3♂♂), at canopy of the same tree (1♂), light, 3.030416, 16.142138, 23–24.X.2008 (PAPC), Coll. Epiphyte 2008 Team; Dzanga-Ndoki National Park, base camp, Lake #1, at the base of an Azobé (*Lophira alata*, Ochnaceae), light (3♂♂), 2.480416, 16.215527, 26.XI.2010 (PAPC); Little forest clearing at Lake #5, light (1♂), 2.469055, 16.225583, 29.XI.2010 (PAPC); Base camp, Lake #1, Laboratory tent, diffuse light (3♂♂), 2.480416, 16.215527, 30.XI to 2.XII.2010 (PAPC), Coll. SANGHA2012 Team.

#### Natural history.

According to the collection locations of different individuals in the canopy, this species is considered to be arboreal. Both nymph and adult specimens, are presumed to reside on the inflorescences of trees. In tropical forests, these flowers are often located at the top, above the canopy, so that pollinators have access to pollen and nectar. In the present study, is only males were captured with a light trap, and were rarely captured during the day. Females *Chlidonoptera* specimens that were observed by climbing trees or by beating vegetation (Figure 2).

#### Diagnosis.

Larger than *Chlidonoptera vexillum* and *Chlidonoptera lestoni*. Males: Body length (mm) 26.2–33.6; forewing length 23.6–30.2; hindwing length 24.9–27.3; pronotum length 5.1–6.9; prozone length 2.1–3.5; pronotum width 4.9–6.3; pronotum narrow width 1.6–2.1; head width 5.0–5.9; frons width 1.4–2.0; frons height 0.6–0.9; prothoracic coxae length 6.1–9.0; prothoracic femur length 8.0–10.2; mesothoracic femur length 6.2–8.1; mesothoracic tibia length 5.5–6.9; mesothoracic tarsus length 4.8–6.1; metathoracic femur length 7.2–9.1; metathoracic tibia length 6.5–8.4; metathoracic tarsus length 5.5–6.9; anteroventral femoral spine count 10–12; posteroventral femoral spine count 4; anteroventral tibial spine count 12–15; posteroventral tibial spine count 14–17. The colour patterns on the wings are almost similar (Figures 2–4). There are polymorphisms in the size of the forewings’ patterns in each of the species mentioned. The major difference is in the size of body, of genitalia and of the posterior process of sclerite L4A (ventral phallomere) being larger from one species to another (Figures 5, 6).

#### Description.

**Male.** General colour of the body green and pale yellow. Holotype: Body length (mm) 30.4; forewings length 27.5; hindwings length 25.6; pronotum length 6.3; prozone length 3.0; pronotum width 5.5; pronotum narrow width 2.0; head width 5.8; frons width 1.9; frons height 0.9; prothoracic coxae length 8.1; prothoracic femur length 9.8; mesothoracic femur length 8.0; mesothoracic tibia length 6.5; metathoracic tarsus length 5.2; metathoracic femur length 8.4; metathoracic tibia length 7.7; metathoracic tarsus length 6.2; anteroventral femoral spine count R12/L12; posteroventral femoral spine count R4/L4; anteroventral tibial spine count R13/L14; posteroventral tibial spine count R15/L16.

***Head***: Oval with anteriorly protruding eyes; vertex arcuate with pronounced tubercles at the sides; prolongation of the bifid vertex; lower frons markedly concave, superior margin angles have a tubercle, raised lateral margins; the median region of third antennal segment is black.

***Pronotum***: Presenting no special features in comparison with *C. vexillum* and *C. lestoni*. Pronotum slightly longer and wider than in other species with always two tubercles slightly directed forward, just above the supracoxal sulcus. Crenellated edges with tubercles of variable sizes. Greenish prozone in the centre and whitish on the sides. Green metazone except on the margin.

***Forelegs***: Legs very similar in their morphology and coloration to those of the other species previously cited. The anterior femora always with four discoidal spines, four posteroventral femoral spines, and 10–12 anteroventral femoral spines. Anterior tibia has 12–14 anteroventral tibial spines and 14–17 posteroventral tibial spines.

***Meso- and metathoracic legs***: Legs very similar in their morphology and coloration to those of the other species previously cited.

***Wings***: Forewing 23.6–30.2 mm in length, featuring the usual colour pattern for the genus, with a yellow spot contained between the two black arcs in a relatively large circle. Hindwings 24.9–27.3 mm long, hyaline, with basal region more or less yellow with red-brownish veins.

***Abdomen***: It presents no special features in comparison with *C. vexillum* and *C. lestoni*. Laterally lobed abdominal segments. Subgenital plate more or less asymmetrical as in the other species; supraanal plate and cerci without special features.

***Genitalia***: Same type of *C. vexillum* with the posterior process of the ventral phallomere longer and thicker than in *C. vexillum* and a ventral phallomere longer (Figures 5, 6).

#### Etymology.

This species is named in honour of my oldest daughter, Roxane, who was growing in her mother’s womb, while I was deep in the primary forest of the Central African Republic, for field work in February 2012.

#### DNA barcoding.

**Figure 7.**
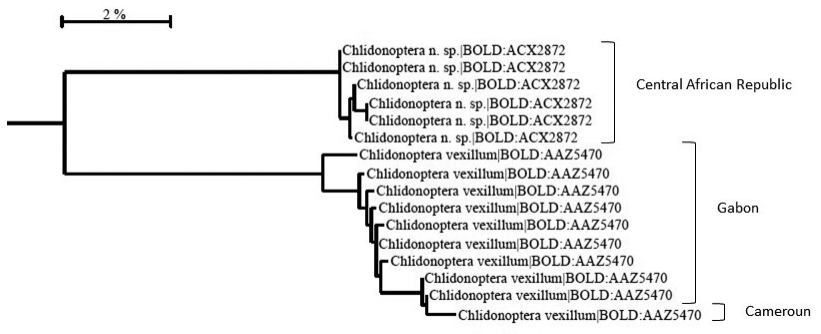
Barcode tree of *Chlidonoptera* from Central Africa created in BOLD using a Neighbour-Joining analysis.

Nineteen sequences were obtained from the 25 specimens sampled (Figure 7). *C. roxanae* sp. nov. and *C. vexillum* are distant enough from each other (9.4% between them), to allow us to consider them as two different species. BINs (Barcode Index Number) have been attributed to them: BIN: BOLD: ACX2872 for *C. roxanae* sp. nov. (mean intraspecific divergence 0.19%) and BIN: BOLD: AAZ5470 for *C. vexillum* (mean intraspecific divergence 0.76%). No fresh specimens of *C. lestoni* were obtained for barcoding. Nuclear mitochondrial pseudogenes (numts) sometimes lead to the creation of different BINs, a problem which was not encountered here with the differences on the genitalia and the larger general morphology. PCR did not work for six specimens, presumably due to their condition, as they had to be relaxed in order to be mounted, or due to the preserving liquid. These specimens came from Cameroon, Gabon, Republic of the Congo, Tanzania, and Uganda.

## Discussion

A larger size, differing genitalia, barcoding analysis, and an isolated geographical location, allowed us to distinguish *C. roxanae* sp. nov. from other *Chlidonoptera* species. Since it was not possible to find the male specimen of *Chlidonoptera* cited in the publication of Roy (2018) about Mantodea in the La Maboké area of the Central African Republic, it is not possible to rule on the species. Geographically, that specimen seems to fit with *C. roxanae* sp. nov., without certainty, like those in the Sangha-Mbaere region (Moulin et al. 2017). For the specimens from Central East Africa (Democratic Republic of the Congo, Tanzania, Uganda), where PCR did not work, fresh material will be required to perform additional barcoding and to confirm a *C. vexillum* identification. It is well-known that DNA barcoding revealed cryptic species of Australian Phasmida among specimens organised at the level of morphospecies (Velona et al. 2015). Molecular analyses are of particular importance for a morphologically conserved group of organisms such as Mantodea (but not between genera) or Phasmida.

## Supplementary Material

XML Treatment for Chlidonoptera

XML Treatment for Chlidonoptera vexillum

XML Treatment for Chlidonoptera werneri

XML Treatment for Chlidonoptera chopardi

XML Treatment for Chlidonoptera lestoni

XML Treatment for Chlidonoptera roxanae
